# Optimal Thyrotropin Suppression Therapy in Low-Risk Thyroid Cancer Patients after Lobectomy

**DOI:** 10.3390/jcm8091279

**Published:** 2019-08-22

**Authors:** Yu-Mi Lee, Min Ji Jeon, Won Woong Kim, Tae-Yon Sung, Ki-Wook Chung, Young Kee Shong, Suck Joon Hong

**Affiliations:** 1 Department of Surgery, Asan Medical Center, University of Ulsan College of Medicine, Seoul 138-736, Korea; 2 Department of Internal Medicine, Asan Medical Center, University of Ulsan College of Medicine, Seoul 138-736, Korea

**Keywords:** lobectomy, low-risk thyroid carcinoma, thyrotropin suppression treatment

## Abstract

Background: This study aimed to identify the clinical results after thyrotropin suppression therapy (TST) cessation and evaluated clinical factors associated with successful TST cessation. Methods: Patients who underwent lobectomy due to low-risk papillary thyroid carcinoma (PTC) were included in this study. We compared clinical characteristics and outcomes between patients who succeeded to stop TST and failed to stop TST. Results: A total of 363 patients were included in the study. One hundred and ninety-three patients (53.2%, 193/363) succeeded to stop TST. The independent associated factors for successful TST cessation were the preoperative thyroid-stimulating hormone (TSH) level and the maintenance period of TST. Patients with low TSH level showed a higher success rate for levothyroxine (LT4) cessation than patients with high TSH level (1.79 ± 1.08 and 2.76 ± 1.82 mU/L, *p* < 0.001). Patients who failed to discontinue TST showed a longer maintenance period of TST than patients who succeeded to discontinue TST (54.09 ± 17.44 and 37.58 ± 17.68 months, *p* < 0.001). Conclusions: Preoperative TSH level and maintenance period of TST are important factors for successful cessation of TST. If TST cessation is planned for patients who are taking LT4 after lobectomy, a higher success rate of TST cessation is expected with low preoperative TSH level and early cessation of LT4.

## 1. Introduction

Total thyroidectomy with thyrotropin suppression therapy (TST) is the typical treatment for patients who were treated for differentiated thyroid carcinoma (DTC). The purpose of levothyroxine (LT4) therapy is not only to replace endogenous thyroid hormone but also to prevent relapse or progression of thyroid cancer. The principle behind TST is the use of supraphysiological doses of LT4 to reduce thyroid-stimulating hormone (TSH) [1,2]. TST exerts an inhibitory effect on DTC growth [3,4] because TSH is mandatory for proliferation of DTC cells.

With the improvement in early diagnosis, the proportion of patients with low-risk DTC is also increasing [5,6,7], and conservative treatments are preferred recently. Thyroid lobectomy has already become the most preferred surgical method for patients with low-risk thyroid cancer, and the role of TST in patients who underwent lobectomy needs to be reconsidered, accordingly. Unlike the 2009 guidelines that recommended a strict TST policy to all patients [8], the 2015 revised guidelines of the American Thyroid Association (ATA) recommended a modest degree of TSH suppression to patients with low-risk DTC [9]. Moreover, they suggested that TST might not be needed if patients who underwent lobectomy could maintain their serum TSH at the lower limit of the normal range (0.5–2.0 mU/L).

However, there is no consensus on how to manage low-risk DTC patients who have already started TST according to the previous guidelines. TST cessation may sometimes be considered after 5 or 10 years depending on the experience of the clinician, but many of the patients who underwent lobectomy routinely maintain LT4 for their lifetime. Considering the excellent outcome of low-risk DTC and potential adverse effects of TST [10], it is not appropriate to use lifelong LT4 for all the patients with low-risk DTC who underwent lobectomy.

This study aimed to identify the clinical results after TST cessation and evaluated clinical factors associated with successful TST cessation. We compared clinical characteristics and outcomes between patients who succeeded to stop TST and failed to stop TST. Then, we evaluated the success rate of TST cessation and clinical factors associated with LT4 cessation.

## 2. Materials and Methods

### 2.1. Patients and Study Design

**Figure 1:**
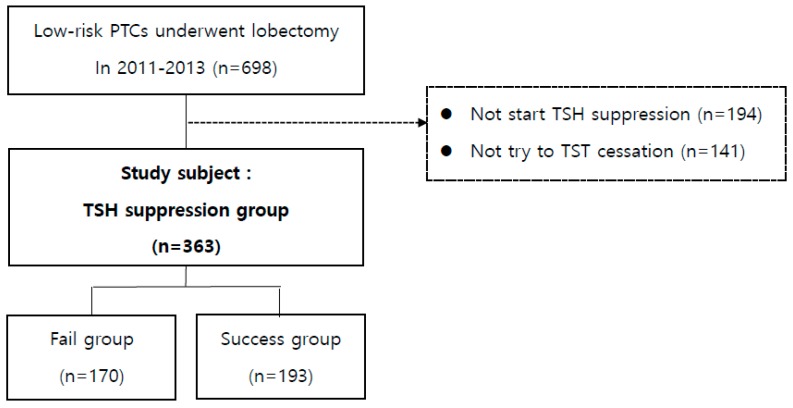
Study design.

A total of 2462 patients underwent lobectomy due to papillary thyroid carcinoma (PTC) at Asan Medical Center (Seoul, Korea) from 2011 to 2013, and there were 698 patients who underwent lobectomy due to low-risk PTC. The baseline characteristics of patients with indeterminate or high-risk groups who were excluded from this study were summarized at Supplement Table S1. Among these patients, we excluded the patients who never started TSH suppression (194 patients) and who did not try to TST cessation (141 patients). Finally, a total of 363 patients were included in the analysis. These patients were then classified into two groups according to whether they succeeded to discontinue TST or not—success group and fail group (Figure 1).

Data were obtained from the prospectively maintained endocrine surgery database available at Asan Medical Center. The study protocol was approved by Asan Medical Center Institutional Review Board and the requirement for informed consent from each patient was waived because of the non-interventional nature of the study.

### 2.2. Initial Treatment and Follow-Up Protocol

Thyroid lobectomy was recommended only for patients with low-risk DTC based on the ATA guidelines. Preoperative neck ultrasonography (US) was performed in all patients to examine the contralateral lobe and cervical lymph node (LN). If the US indicated the presence of suspicious thyroid nodules in the contralateral lobe or cervical LN metastasis in the lateral neck, US-guided fine-needle aspiration cytology (FNAC) specimen was obtained to examine the lesions prior to thyroid surgery. Prophylactic unilateral central neck dissection was routinely performed for patients who underwent lobectomy.

We have not usually used TST to patients who underwent lobectomy due to low-risk DTC since 2011. Some patients underwent TST with LT4; the decision for TST after lobectomy was determined by clinical risk factors such as age, sex, tumor size, cervical LN metastasis, and preference of the endocrinologist and patients. Even in these patients who underwent TST, LT4 cessation was considered actively when they had normal thyroid function and maintained a low thyroglobulin (Tg) level with undetectable Tg antibody (TgAb). We offered information about the advantages and disadvantages of TST to such patients and then tried to discontinue TST under their agreements.

Patients taking ≥100 µg of LT4 tapered their medication by 25–50 µg every 3 months, then they stopped LT4 when 25–50 µg of dose was reached. If the thyroid function test maintained a euthyroid status without LT4 until the last follow-up, TST cessation was considered successful (success group). In patients with high-normal thyroid function (TSH 2.0–5.0 mIU/L) or subclinical hypothyroidism (TSH 5.1–10.0 mIU/L) without symptoms, short-term follow-up (2–3 months) without LT4 was carried out, instead of immediate reuse of LT4. If overt hypothyroidism (TSH > 10.0 mIU/L) developed after reducing/discontinuing the drug or the patients complained of symptoms of hypothyroidism, the dose of LT4 was increased or LT4 was restarted. In those cases, such patients were considered in the “fail group,” in which TST cessation failed.

All patients underwent follow-up examinations at an outpatient clinic. Thyroid function tests, which included serum TSH, serum-free thyroxine and Tg, were routinely performed every 6 months, and neck US was performed once a year. Any patients suspected to have locoregional recurrence underwent US-guided FNAC. Distant metastasis was diagnosed using whole-body scan, chest computed tomography (CT), or 18-fluorodeoxyglucose positron emission tomography/CT and confirmed using serial imaging or biopsy.

Unfortunately, our country’s health insurance does not allow tests for thyroid antibodies, such as thyroid peroxidase (TPO) antibody, to be performed. Hence, we could not diagnose thyroiditis through serologic tests. In this study, thyroiditis was only defined as lymphocytic thyroiditis on the final pathologic reports after surgery. Structural recurrence was defined as the appearance of cytologically or histopathologically proven malignant tissue or the appearance of highly suspicious structural lesions on cross-sectional or functional imaging studies. Biochemical recurrence, characterized by an elevated serum Tg level without clinical evidence of structural disease, was not classified as true recurrence.

### 2.3. Statistical Analysis

Continuous variables are presented as means ± standard deviations or as medians and ranges, whereas categorical variables are presented as percentages and absolute numbers. Continuous variables were examined using Student’s *t*-test and Wilcoxon’s rank sum test, whereas categorical variables were analyzed using the chi-square test or Fisher’s exact test. Logistic regression model was used to identify the associated factors for TST cessation. Odds ratios with 95% confidence intervals (CIs) were calculated. All clinic-pathological variables were entered into the univariate and multivariate analyses. For clinical applicability, some continuous variables among the independent factors as proven by multivariate analysis were converted into categorical variables with cut-off values and area under the curve calculated by receiver-operating characteristic curve analysis based on log-rank tests. All *p*-values were two sided, with values <0.05 considered statistically significant. Statistical analyses were performed using IBM SPSS Statistics for Windows (version 22.0, IBM, Armonk, NY, USA).

## 3. Results

### 3.1. Baseline Patient Characteristics

The baseline characteristics of 363 patients are presented in Table 1. The median follow-up duration was 67 (range, 25–97) months. Only one patient developed recurrence during this period, and she showed contralateral lobe recurrences. There was no patient who died during the follow-up period.

### 3.2. Clinical Factors Associated with Successful TST Cessation

Among 363 patients who had tried to stop TST, 193 patients (53.2%) succeeded to discontinue LT4. Analysis of clinical features associated with successful TST cessation revealed the preoperative TSH level and maintenance duration of TST (Table 2). Patients who failed to stop TST showed a higher preoperative TSH (2.76 ± 1.82 mIU/L) level and longer maintenance period of TST (54.09 ± 17.44 months) than patients who succeeded to stop TST (preoperative TSH, 1.79 ± 1.08 mIU/L; maintenance period of TST, 37.58 ± 17.68 months; *p* < 0.001, both). By multivariate analysis, they were also the independent factors associated with successful TST cessation. Table 3 showed the analysis of these independent factors using their cut-off values.

### 3.3. Success Rate of TST Cessation According to the Timing of LT4 Cessation

**Figure 2:**
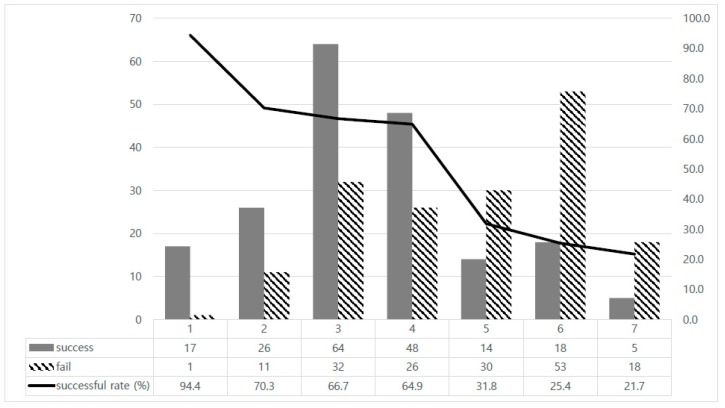
Success rate of levothyroxine cessation according to timing.

When the success rate of TST cessation was compared with the timing of LT4 cessation, patients with long maintenance period of TST showed lower success rate of TST cessation (Figure 2). In patients who stopped LT4 within one year after surgery, most of them (17/18, 94.4%) could maintain euthyroid status without LT4. About 68.9% (155/225) of patients recovered normal thyroid function after LT4 cessation when they had stopped LT4 before five years, whereas 26.8% (37/138) of patients successfully stopped LT4 among those who maintained LT4 over five years.

## 4. Discussion

In this study, preoperative TSH level and maintenance period of TST are the important factors associated with the success rate of TST cessation. Patients with low TSH level showed a higher success rate for LT4 cessation than patients with high TSH level. As the duration of LT4 maintenance increased, the success rate of LT4 cessation decreased. In particular, among patients who took LT4 for more than five years, about 30% of them were able to recover their normal thyroid function after LT4 cessation.

Low-risk DTC has surely an excellent prognosis, and our study also proved that. There was only one patient (0.3%) who showed local recurrence and after the second surgery, she has maintained a disease-free status. Previous studies have reported that the suppression of serum TSH levels could reduce cancer-specific mortality or disease recurrence, especially in high-risk patients [11,12,13,14,15]. They agreed that extreme low TSH suppression could give better prognosis to patients with high-risk DTC. However, controversy about the efficacy of TST in patients with low-risk DTC still remains. Even in the studies that demonstrated the effect of TST, many of them failed to show any impact of TST on the patients with low-risk DTC [11,13]. One study has recently reported that TST showed a limited benefit on prognosis even in the indeterminate- or high-risk DTC patients [16]. Therefore, in the era of emphasizing importance of a risk-adapted approach and individualized treatment, it is not appropriate to perform TST uniformly to all patients with low-risk DTC. In the 2015 revised guidelines of the ATA [9], they recommended a more conservative management for patients with low-risk DTC.

A Japanese study proposed a more active suggestion [17]. This prospective study reported no significant differences in the five-year disease-free survival (DFS) according to TSH suppression for patients with high- and low-risk PTC. They suggested that thyroid-conserving surgery without TSH suppression should be considered for patients with low-risk PTC. Previous research from our institute also showed similar results [18]. After propensity matching analysis of patients who underwent lobectomy for ATA low-risk DTC, we found that there was no difference in DFS and similar dynamic risk stratification between the TST and non-TST groups. In addition, no significant difference was observed in the DFS between both groups by comparing serum TST levels.

Through these evidence and clinical experiences, we have performed TST use differently according to patients’ clinic-pathological features and this policy has been maintained in our institute since 2011. To patients who underwent lobectomy for low-risk DTC, we did not recommend TST routinely. Even in patients who were prescribed LT4 because of various reasons, we attempted to stop TST actively. There were differences in the timing of TST cessation because of preference of the endocrinologists and patients, and this allowed us to perform this study. We collected and analyzed our data from 2011 to 2013 in order to get enough number of patients with a sufficient follow-up period facilitating statistical analysis.

In our study, one of the associated factor to successful LT4 cessation was preoperative TSH level. Many studies reported that preoperative TSH level was an important risk factor for hypothyroidism after lobectomy [19,20,21,22,23]. Higher preoperative TSH level might reflect decreased functional reserves in the thyroid gland. In the current study, we found that preoperative TSH level with the cut-off value of 1.95 mIU/L could be an indicator for recovery of thyroid function after LT4 cessation. We expect this to be useful in clinical field when planning TST cessation.

In the situation of no evidence about TST maintenance period in low-risk patients who underwent lobectomy, our study showed an interesting result. The longer that LT4 was used, the more difficult it was to stop LT4. Most patients who tried to stop within one year could obtain a euthyroid status, whereas those who maintained LT4 for more than one year had a higher rate of re-taking LT4 because of impaired thyroid function. Intriguingly, among patients who had taken LT4 for more than five years, one third of them could discontinue TST, and it was less than half the success rate compared to that of the patients who discontinued LT4 before 5 years.

Some studies demonstrated that long use of LT4 could inhibit the function of recoverable thyroid gland [20,21,22]. Although the mechanism has not been clearly understood, suppressed TSH level by exogenous LT4 might result in decreased activity of pituitary thyrotropin or TSH receptors of remnant thyroid tissue. This could be a permanent suppression of pituitary thyrotropin or TSH receptor if TSH suppression was kept for a long period, and then eventually lead to permanent hypothyroidism. Our study showed that more than five years of LT4 use affected adversely the function of remnant thyroid tissue. Therefore, when TST cessation was planned in low-risk DTC patients who used LT4 according to the previous guidelines, TST should be stopped as soon as possible.

This study has several limitations. First of all, we did not have information on TPO antibody, because of restriction of our country’s health insurance. Consideration of the relation of TPO antibody to thyroiditis and thyroid dysfunction could be one of the limitations of our study. In addition, this retrospective cohort study has a potential selection bias. Although patients were followed according to institutional protocols, the schedule of thyroid function measurements might have differed between patients. To overcome these limitations, the present study only enrolled patients who strictly met the inclusion criteria. Unfortunately, this policy resulted in the exclusion of many patients, and this became one of the causes of insufficient statistical analysis power. Therefore, studies with long-term follow-up, standardized discontinuation schedules, and larger and more diverse patient populations should be required. However, this was a unique study evaluating the results of TST cessation in low-risk PTC patients who underwent lobectomy with a relatively large number of patient cohorts and long-term follow-up period.

## 5. Conclusions

Considering the excellent prognosis of low-risk DTC and limitation of TST effect, TST is not necessary for patients who undergo lobectomy for low-risk DTC. In addition, it is needed to consider to stop LT4 to low-risk DTC patients who have already started TST according to the previous guidelines. Preoperative TSH level and maintenance period of TST are the important factors for a successful cessation of TST. If TST cessation is planned for patients who are taking LT4 after lobectomy, a higher success rate of TST cessation is expected with low preoperative TSH level and early cessation of LT4.

## Figures and Tables

**Table 1:** Baseline characteristics of patients with low-risk papillary thyroid carcinoma who underwent lobectomy and received TST.

	Total Patients (*n* = 363)
Age (years, mean ± SD)	52.21 ± 9.88
Sex	
Male	67 (18.5%)
Female	296 (81.5%)
Tumor size (cm, mean ± SD)	0.59 ± 0.30
Thyroiditis	
No	260 (71.6%)
Yes	103 (28.4%)
Multifocality	
No	305 (84.0%)
Yes	58 (16.0%)
N stage	
N0	279 (76.9%)
N1a	84 (23.1%)
Recurrence	
No	362 (99.7%)
Yes	1 (0.3%)

SD, standard deviation; TST, thyrotropin suppression therapy.

**Table 2:** Univariate and multivariate analysis for clinical factors associated with successful cessation of thyrotropin suppression therapy.

	Fail *n* = 170 (46.8%)	Success *n* = 193 (53.2%)	Univariate		Multivariate	
			OR (95% CI)	*p* Value	OR (95% CI)	*p* Value
Age (years, mean ± SD)	51.67 ± 9.72	52.70 ± 10.02	1.01 (0.99–1.03)	0.319	1.01 (0.99–1.04)	0.395
Sex				0.709		0.459
Male	30 (17.6%)	37 (19.2%)	Ref.		Ref.	
Female	140 (82.4%)	156 (80.8%)	0.90 (0.53–1.54)		0.78 (0.41–1.49)	
Tumor size (cm, mean ± SD)	0.59 ± 0.27	0.60 ± 0.33	1.13 (0.57–2.24)	0.727	1.26 (0.57–2.79)	0.574
Multifocality				0.519		0.510
No	119 (70.0%)	141 (73.1%)	Ref.		Ref.	
Yes	51 (30.0%)	52 (26.9%)	0.86 (0.55–1.36)		0.80 (0.41–1.56)	
Thyroiditis				0.739		0.738
No	144 (84.7%)	161 (83.4%)	Ref.		Ref.	
Yes	26 (15.3%)	32 (16.6%)	1.10 (0.63–1.94)		1.11 (0.60–2.04)	
N stage				0.507		0.676
N0	128 (75.3%)	151 (78.2%)	Ref.		Ref.	
N1a	42 (24.7%)	42 (21.8%)	0.85 (0.52–1.38)		1.13 (0.64–2.00)	
Preoperative TSH (mIU/L, mean ± SD)	2.76 ± 1.82	1.79 ± 1.08	0.58 (0.48–0.71)	<0.001	0.61 (0.50–0.75)	<0.001
TSH on trying to stop TST (mIU/L, mean ± SD)	0.90 ± 0.82	0.96 ± 0.98	0.86 (0.86–1.35)	0.535	1.03 (0.78–1.35)	0.845
Maintenance period of TST (months, mean ± SD)	54.09 ± 17.44	37.58 ± 17.68	0.95 (0.94–0.96)	<0.001	0.95 (0.94–0.97)	<0.001

CI, confidence interval; OR, odds ratio; Ref., reference; SD, standard deviation; TSH, thyrotropin; TST, TSH suppression therapy.

**Table 3:** Independent clinical factors associated with successful cessation of thyrotropin suppression therapy and theirs results using cutoff values.

	Fail	Success	Odds Ratio (95% CI)	*p* Value
Preoperative TSH (mIU/L)				<0.001
≤1.95* (*n* = 184, 50.7%)	65 (35.3%)	119 (64.7%)	Ref.	
>1.95* (*n* = 179, 49.3%)	105 (58.7%)	74 (41.3%)	0.18 (0.12–0.29)	
Maintenance period of TST (months)				0.001
≤41 ^†^ (*n* = 185, 51.0%)	49 (26.5%)	136 (73.5%)	Ref.	
>41 ^†^ (*n* = 178, 49.0%)	121 (68.0%)	57 (32.0%)	0.44 (0.28–0.70)	

CI, confidence interval; Ref., reference; TSH, thyrotropin; TST, TSH suppression therapy. * *p* < 0.05, the optimum cut-off point for preoperative TSH was 1.95 mIU/L (area under the curve = 0.69) by using receiver operating characteristic curve analysis. ^†^ *p* < 0.05, the optimum cut-off point for maintenance period of TST was 41 months (area under the curve = 0.74) by using receiver operating characteristic curve analysis.

## Supplementary Materials

The following are available online at https://www.mdpi.com/2077-0383/8/9/1279/s1, Table S1: Baseline characteristics of patients who excluded from this study.
