# Supplementary material for: Optimal Thyrotropin Suppression Therapy in Low-Risk Thyroid Cancer Patients after Lobectomy
Source: J Clin Med. 2019 Aug 22;8(9):1279. doi: 10.3390/jcm8091279 (PMC6780946; doi:10.3390/jcm8091279)
Supplement: Supplementary file 1 [file jcm-08-01279-s001.pdf]

Journal of Clinical Medicine

Supplement Table S1. Baseline characteristics of patients who excluded from this study.

	**Total patients (*n* = 1764)**
Age (years, mean±SD)	45.91 ± 11.92
Sex	
Male	219 (12.4%)
Female	1,545 (87.6%)
Tumor size (cm, mean±SD)	1.76 ± 1.34
Thyroiditis	
No	1,167 (66.2%)
Yes	597 (33.8%)
Multifocality	
No	1,229 (69.7%)
Yes	535 (30.3%)
N stage	
N0	390 (22.1%)
N1a	1,374 (77.9%)
ATA risk	
Indeterminate	1,607 (91.1)
High	157 (8.9)
Recurrence	
No	1,581 (89.6%)
Yes	183 (10.4%)

ATA, America Thyroid Association; D, standard deviation.
